# Results of ultrasound-assisted brace casting for adolescent idiopathic scoliosis

**DOI:** 10.1186/s13013-017-0130-2

**Published:** 2017-08-08

**Authors:** Edmond H. Lou, Doug L. Hill, Andreas Donauer, Melissa Tilburn, Douglas Hedden, Marc Moreau

**Affiliations:** 1 Department of Surgery, University of Alberta, 6-110F, Clinical Science Building, 8440-112 Street, Edmonton, Alberta T6G 2B7 Canada; 2 Department of Research and Innovation Development, Glenrose Rehabilitation Hospital, Edmonton, Alberta T5G 0B7 Canada; 3 Department of Prosthetics and Orthotics, Glenrose Rehabilitation Hospital, Edmonton, Alberta T5G 0B7 Canada

**Keywords:** Adolescent idiopathic scoliosis, 3D ultrasound imaging, Brace treatment, Brace design, Optimum brace pressure

## Abstract

**Background:**

Four factors have been reported to affect brace treatment outcome: (1) growth or curve based risk, (2) the in-brace correction, (3) the brace wear quantity, and (4) the brace wear quality. The quality of brace design affects the in-brace correction and comfort which indirectly affects the brace wear quantity and quality. This paper reported the immediate benefits and results on using ultrasound (US) to aid orthotists to design braces for the treatment of scoliosis.

**Methods:**

Thirty-four AIS subjects participated in this study with 17 (2 males, 15 females) in the control group and 17 (2 males, 15 females) in the intervention (US) group. All participants were prescribed full time TLSO, constructed by either of the 2 orthotists in fabrication of spinal braces. For the control group, the Providence brace design system was adopted to design full time braces. For the intervention group, the custom standing Providence brace design system, plus a medical ultrasound system, a custom pressure measurement system and an in-house software were used to assist brace casting.

**Results:**

In the control group, 8 of 17 (47%) subjects needed a total of 11 brace adjustments after initial fabrication requiring a total of 28 in-brace radiographs. Three subjects (18%) required a second adjustment. For the US group, only 1 subject (6%) required adjustment. The total number of in-brace radiographs was 18. The *p* value of the chi-square for requiring brace adjustment was 0.006 which was a statistically significant difference between the two groups. In the intervention group, the immediate in-brace correction as measured from radiographs was 48 ± 17%, and in the control group the first and second in-brace correction was 33 ± 19% and 40 ± 20%, respectively. The unpaired 2 sided Student’s *t* test of the in-brace correction was significantly different between the US and the first follow-up of the control group (*p* = 0.02), but was not significant after the second brace adjustment (*p* = 0.22).

**Conclusions:**

The use of the 3D ultrasound system provided a radiation-free method to determine the optimum pressure level and location to assist brace design, resulting in decreased radiation exposure during follow-up brace evaluation, increased the in-brace correction, reduced the patients’ visits to both brace adjustment and scoliosis clinics. However, the final outcomes could not be reported yet as some of patients are still under brace treatment.

**Trial registration:**

NCT02996643, retrospectively registered in 16 December 2016

## Background

Adolescent idiopathic scoliosis (AIS) is a three-dimensional deformity of the spine associated with vertebral rotation due to an unknown cause. It is a chronic and a potentially progressive spinal deformity affecting 2–3% of the population [1]. Girls tend to progress more often than boys [2]. Although scoliosis is rarely life threatening, the long-term impact of untreated scoliosis is still controversial [3–8]. Patients with untreated curves usually have more back pain [2, 5], loss of function, external deformity, poor self-image, and in more severe cases, can impair respiratory capacity later in their life. Bracing is typically prescribed either based on guidelines set by the Scoliosis Research Society [9] or by the Society on Scoliosis Orthopaedic and Rehabilitation Treatment (SOSORT) [10], in which the Cobb angle is greater than 20° with considerable growth remaining or show at least 5^o^ of Cobb angle increase between consecutive clinic visits. Recent scientific evidence has shown that brace treatment is effective [11–14], and a pilot study from a single centre has shown a predicted success rates of 95%, when brace wear quantity combined with the brace wear quality is over 43% of the prescribed dosage [15]. A combined value of brace wear quantity and quality can be achieved in many different ways by trading off wear time and wear tightness; a subject can wear the brace 43% of prescribed time (9.9 h/day) and 100% of time at the prescribed tightness level. Similarly, when a subject wears a brace 100% of prescribed time (23 h/day), but only 43% of time at the prescribed level, the subject may get a similar result. Besides these two factors, the (a) growth or curve based risk and (b) the in-brace correction [16, 17] also affect brace treatment outcomes. The curve-based risk is estimated by physical maturity, gender, the severity and location of the curve, and the spinal balance. The in-brace correction may be affected by the brace design and spinal flexibility.

A typical spinal brace is a hard plastic shell with pads installed inside the liner to concentrate and direct the corrective pressure to oppose the spinal curvature. However, the locations of pads are set empirically based on guidelines for the type of the brace or knowledge derived from orthotists’ experiences. Suboptimal pad placement and applied pressure will reduce the in-brace correction which is typically reviewed 6 weeks after the brace has been initiated. If the in-brace correction is not deemed to be satisfactory by the treating orthopedic surgeon, the patient returns to the orthotist for readjustment. This adjustment is required because there is no real time feedback provided to the orthotist during the brace design and construction stage. The standard of care requires the use of radiographs to check the in-brace correction. Radiographs are not taken during brace design and construction to minimize radiation exposure to growing children because of the increased risk of cancer. Unfortunately, after the adjustment, the in-brace correction examination is often required again which increases cumulative radiation exposure and shortens effective brace usage.

Although finite element (FE) models have been developed to determine optimal orientations and load magnitudes of pressure pads for brace design [18, 19], these still have practical limitations [20] with evaluation of the brace correction not available until the in-brace follow-up clinic. Recently, ultrasound (US) imaging, a real-time non-invasive and non-ionizing method, was demonstrated to be successful in measuring proxy Cobb angles, vertebral rotation, and flexibility [21–27]. The proxy Cobb angles which use vertebrae lamina positions rather than end plates, measured from ultrasound images have high intra- and inter-reliability as well as correlate well with radiographic measurements [22, 26]. Furthermore, there were studies applying ultrasound to determine the optimum location of the major brace pad [28, 29], but their approach did not provide real-time feedback nor determine the optimum pad pressure. Their ultrasound data were processed between the time the patient had their brace fitting and were returned to receive the modified brace. Researchers were also able to use ultrasound to investigate the time lag between application of spinal orthosis and its effect on scoliotic curvature [30]. Therefore, a clinical trial using ultrasound to assist orthotists to determine optimum pad pressure level and location during the brace design stage was conducted. This paper reports the immediate results obtained from this clinical trial.

## Methods

### Patients

Seventeen consecutive AIS subjects (2 males, 15 females; age 13.2 ± 1.5 years, Cobb 32 ± 9°), with retrospectively collected data who were prescribed a new full time TLSO between January and June 2013 and met the inclusion criteria, served as the control group to match the intervention group recruitment. The distribution of the primary curve of the control group was 7 major thoracic, 6 thoracolumbar, and 4 lumbar curves. Another 17 new AIS subjects (2 male, 15 female; age 13.2 ± 1.4 years, Cobb 35 ± 8°), who were prescribed a TLSO were prospectively recruited between January 2014 and April 2015 into the intervention group. There was no significant difference of the Cobb angle between groups. The distribution of the primary curve of the intervention group was 6 major thoracic, 6 thoracolumbar, and 5 lumbar curves. Local ethics approval (Pro00028133) was granted by the local institution ethics board and all subjects signed consent forms before participation. The inclusion criteria followed the guidelines set by the non-operational management committee of the Scoliosis Research Society [9] (a) age 10 years or older when brace is prescribed, (b) Risser 0–2, (c) primary curve angles 20°–45°, (d) no prior treatment, and (e) if female, either pre-menarchal or less than 1 year post-menarchal. Both participating orthotists are aligned with the same pediatric scoliosis program and worked together using the same methodology to design spinal brace for over 10 years. There was no change on the X-ray system and the clinical protocol during the entire recruitment period (January 2013–April 2015).

### Control group protocol

For the control group, the traditional plaster cast and molded method with the assistance of the Providence brace system to design spinal braces was used. The orthotist first reviewed the standing posteranterior pre-brace radiograph to identify the location of the apices. He/she then applied a plaster rigid wrap to the AIS body while the subject is standing and instructed the subject to lay upon the Providence brace system. The orthotist used the bolsters to apply pressures and adjusted the pressure level based on the location of the curve apex and his/her experience. After the plaster hardened, the subject stood up again to remove the hardened cast. Reflective markers were then placed around the cast and then scanned by a handheld laser scanner to create a 3D casting image file. The 3D file was then imported into software that was linked to a carving machine. Some minor adjustment was done at this stage to smooth the surface. A 3D body mold was then carved using foam material. After subjective modifications for improved fitting and comfort on the foam positive mold, a brace was fabricated. Subjects typically returned to the orthotist to fit the brace and make the final adjustments within a week. After that, the subject would use the brace for about 6 weeks, slowly building up their wear time, and returned to the scoliosis clinic to evaluate the design of the brace primarily based on wearability and the correction obtained from the in-brace radiograph.

### Intervention group protocol

**Fig. 1:**
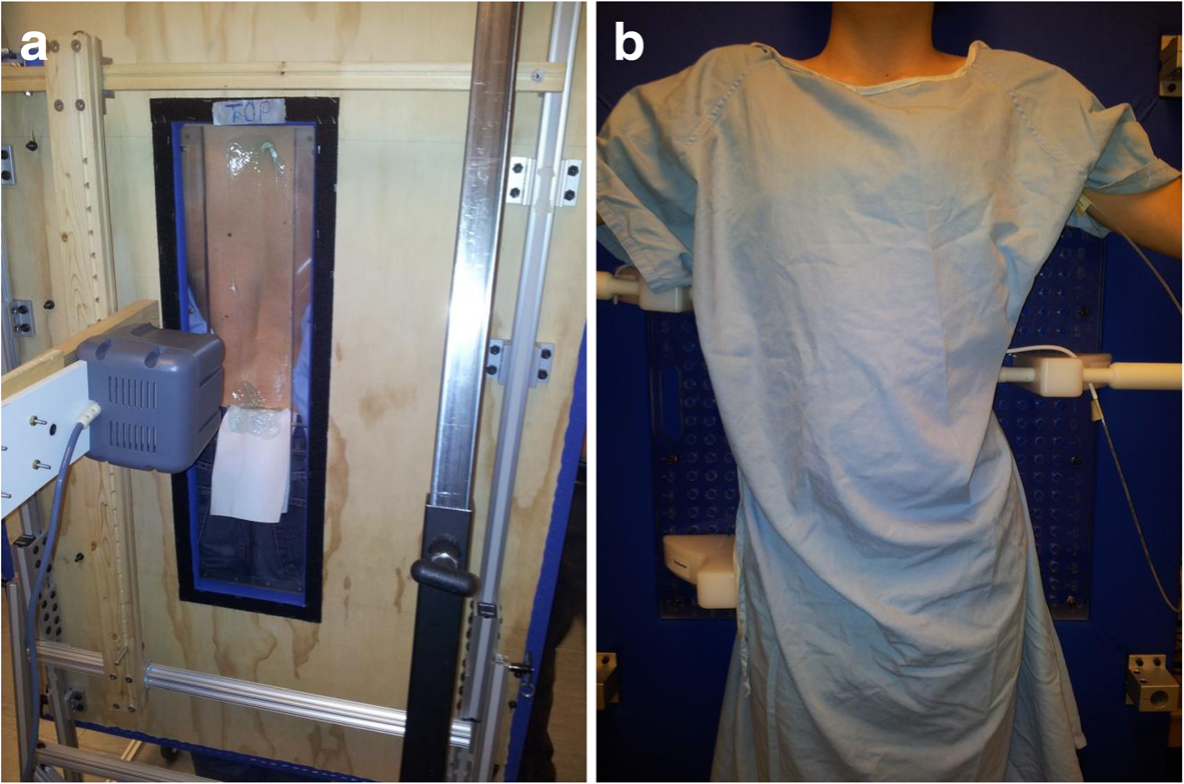
**a** The opening at the back of the frame, and **b** a subject stands on a frame with a custom Providence brace design system

**Fig. 2:**
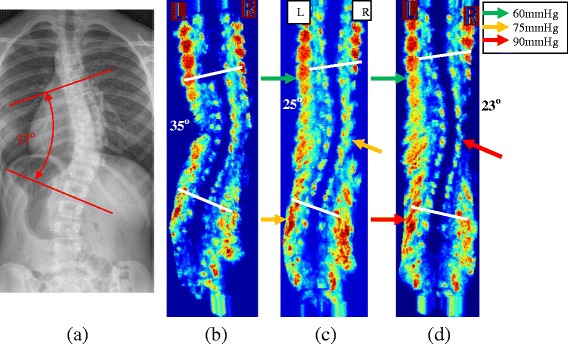
**a** The standing pre-brace X-ray with Cobb angle 37°. **b** The baseline US scan (Cobb angle 35°). **c** The first trial US scan (Cobb angle 25°). **d** The 2nd trial US scan (Cobb angle 23°)

**Fig. 3:**
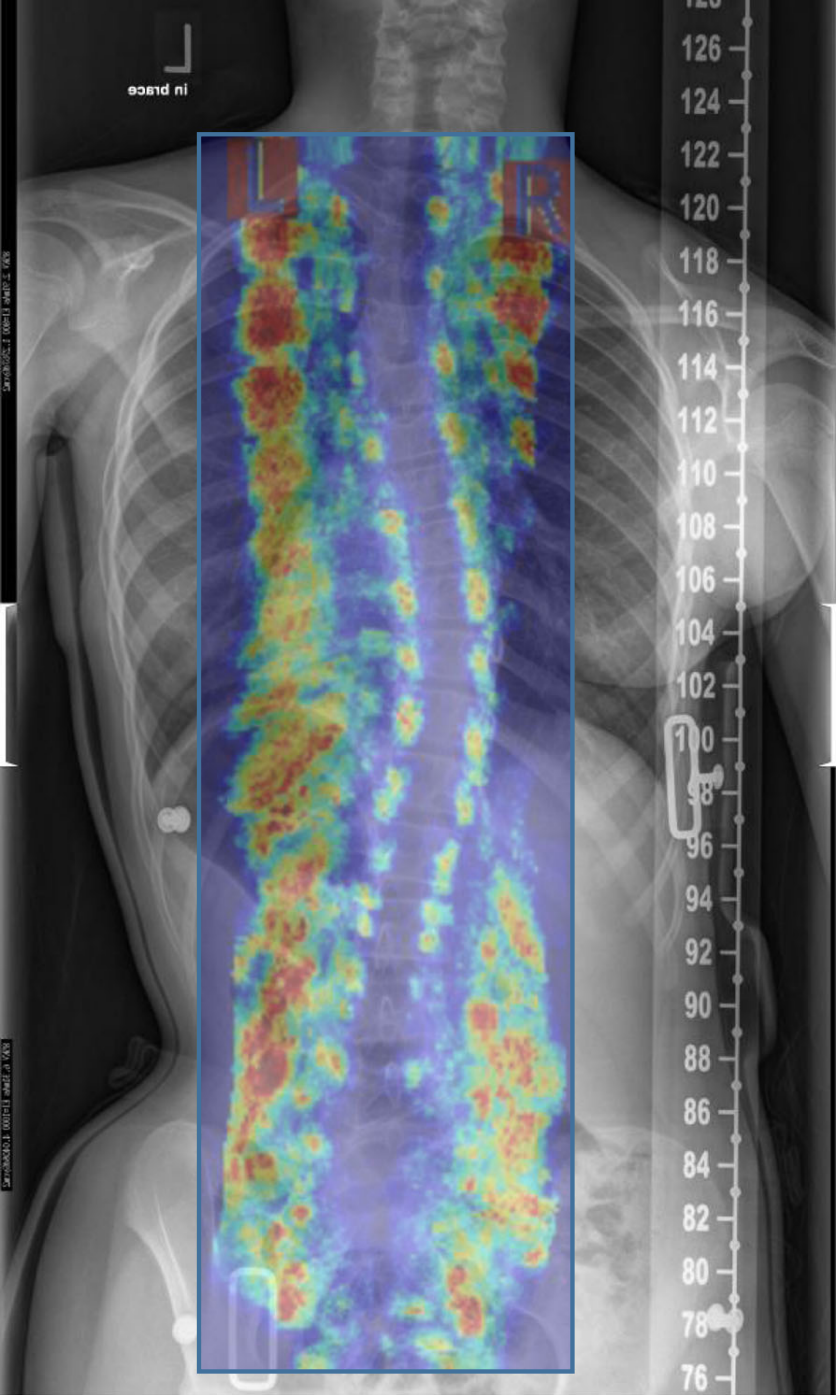
The second US trial overlapped with the in-brace radiograph in which the Cobb angle from the radiograph was 21°

A custom Providence brace standing frame, a medical ultrasound (US) system, a custom pressure measurement system, and in-house US measurement software were used to assist brace casting for the intervention group. A 14 cm × 50 cm opening was cut at the middle of the Providence frame to allow for the ultrasound scanning probe. Figure 1 shows the back of the frame and the custom Providence brace design set up with a subject. The subject wore a gown and stood against the standing frame. An operator with several years US scanning experience scanned the subject using the US system. It took approximately 1.5 min to acquire, process, and display the image. The pre-brace X-ray and the standing pre-pressure US spinal image were displayed side-by-side to assist the orthotist to decide on pressure pads locations. The orthotist used the custom standing Providence brace design system to secure bolsters with subjectively determined applied pressure levels against the patient’s torso to simulate in-brace correction. At each bolster, an air bag was attached on the surface to measure the interface pressure applied between the bolster and body. The simulated in-brace US scan was then acquired. A real-time US spinal image was displayed and the proxy Cobb angles were measured using in-house developed software. This process took less than 2 min. The difference of the ultrasound measurements compared to the corresponding radiographic measurements was 2–3° with good consistency [22]. The orthotist then decided if altering bolster locations and pressure levels might improve correction. Another US scan was taken if the bolster positions were altered. The procedures were repeated until the orthotist attained the best simulated in-brace correction configuration. The target goal was still to try to get at least 50% correction. During scanning, the pressure levels at each bolster were recorded. Figure 2 shows (a) the pre-brace standing X-ray with a right thoracic curve of 37^o^ between T8 and T12, (b) the standing baseline US image with proxy Cobb angle 35^o^, (c) the first US scan with axilla, thoracic, and lumbar pads pressure levels at 60, 75, and 75 mmHg, respectively, at which the Cobb angle is 25^o^, (d) the second US scan with axilla, thoracic, and lumbar pads pressure levels at 60, 90, and 90 mmHg, respectively at which the Cobb angle was 23^o^. The location of each bolster relative to the waist level was recorded. The orthotist then applied a plaster rigid wrap and identical pressure levels to the subject to the best stimulated in-brace correction configuration on a supine position with the Providence system. The pads’ positions and pressure levels recorded from the standing frame were applied. After the plaster hardened and was removed, the cast was scanned by a handheld laser scanner to create a positive mold which was used for brace fabrication. Figure 3 shows the US second trial image overlapped with the in-brace radiograph at which the Cobb angle from the in-brace radiograph was 21^o^.

### First follow-up clinic

Approximately, 6 weeks after braces initiation, all subjects returned to scoliosis clinics to inspect the effectiveness of the brace based on the in-brace correction. The treating orthopedic surgeons used the target threshold of in-brace Cobb correction of 50%. They also used their clinical experience to consider whether the in-brace correction was optimal because the target threshold may not be attainable for rigid curves. If the surgeon was not satisfied with the in-brace correction, the subject would return to the orthotist for adjustments. Ultrasound was not used to assist in the adjustment for either group. Additional follow-up clinic visits with radiographs occurred approximately 2 months after adjustments.

## Results

In the 17 control subjects, the major pre-brace Cobb angle was 32^o^ ± 9^o^. Eight of these required brace adjustment (47%) and 3 of these adjusted subjects (38%) requiring a second adjustment. A total of 11 brace adjustments were needed and 28 in-brace radiographs were taken (average 1.6 radiographs per subject). The average in-brace major Cobb angle correction at the first in-brace follow-up clinic and at the final accepted follow-up clinic were 33 ± 19% and 40 ± 20%, respectively.

For the intervention group, the major pre-brace Cobb angle from the radiographs prior to bracing was 35^o^ ± 8^o^. Only 1 subject (6%) required adjustment. A total of 18 in-brace radiographs were taken (average 1.1 radiographs per subject). The orthotist was satisfied with the first attempt with the US information in 8 out of 17 cases. With 9 subjects, the location and pressure level of the bolsters were altered one time. Among these 9 revised cases, 7 showed better stimulated in-brace corrections, 1 had no change, and 1 got worse. The intervention resulted in 7 out of 17 subjects (42%) having their brace designed using an improved pressure level and/or pad placement. For the 7 improved cases, the in-brace Cobb correction from the US measurements in the first and second trials were 29 ± 11% and 42 ± 14%, respectively. For the intervention group as a whole, the average final in-brace Cobb angle was 19^o^ ± 8^o^ which was 48 ± 17% in-brace correction, which was slightly higher than the simulated US in-brace correction.

The *p* value of the chi-square for requiring brace adjustment between the control and the intervention groups was 0.0065 which was a statistically significant difference. The *p* values of the unpaired two sided Student’s *t* test of the in-brace correction between the two groups were 0.02 and 0.22 between the first and second time of adjustment, respectively. It showed statistically significant difference between the US and the first time for the control group, but no statistically significant difference between the US and the second time of the control group. The reduction of the number of in-brace radiographs was large, 18 in-brace radiographs from the US group versus 28 in-brace radiographs from the control group, a saving of 10 radiographs in 17 subjects. Table 1 also shows the comparison of the health system time to cast and make the brace adjustment between the control and the intervention groups; on average an extra 1 h/per subject was needed in the control group. Furthermore, the time that the control and the intervention group received their optimum designed brace after prescription averaged 3.5 ± 1.9 months compared to 2.1 ± 0.5 months. There was a significant delay to start the effective brace treatment between the two groups.

**Table 1:** Comparison of the casting and the brace adjustment time per subject

	Control group	Intervention group
Casting time	17 h (1 h per subject)	20.4 h (1.2 h per subject)
Brace adjustment time	11 h (1 h per adjustment)	1 h
Extra scoliosis clinic	11 h	1 h
Total time	39 h	22.4 h
Health system time per subject	2.3 h	1.3 h

## Discussion

Brace treatment is now generally accepted as a proven effective method to stop the progression of AIS. Besides compliance, a good brace design is vitally important. In current practice, the skill and experience of the orthotist are the major factors which affect the design of the brace. The pressure pads’ levels, locations, and directions are subjectively selected by the orthotist. Without real-time feedback, trial and error in brace design is used. Lack of acceptable in-brace correction may trigger brace adjustment. Even though Li et al. [28, 29] applied the ultrasound method to assist brace fitting by investigating the locations of pressure pads, they did not provide the real-time feedback to the orthotist. They processed the data later to determine the optimum pad location and required patients to have an extra visit to receive the final brace. In this study, the intervention group has 7/17 (42%) that benefitted from having a brace adjustment after the initial setting of the pad placements. Those 7 cases which included 3 thoracic, 2 thoracolumbar, and 2 lumbar cases, did not indicate this method was only beneficial for specific types of curves. However, since the number of cases is still limited, no conclusive statement can be made. The advantage with the intervention group was that the adjustment was made prior to brace fabrication rather than after the first follow-up visit. The compromise between the comfort and treatment outcomes is influenced by how aggressively the orthotist designs the brace. With the immediate feedback, 7 out of 9 cases (80%) showed the revised bolster placements or pressure alterations resulted in better correction than the first trial. This demonstrates how importantly the pressure pads location affects the effectiveness of the brace treatment. The subjects are able to report their pressure tolerance level that they feel in real-time. Requiring brace adjustment increases not only the number of radiographs and the cost of the health care system (orthotists’, surgeons’ and clinics time), but also the burden for the families that they need to travel to both brace adjustment and extra follow-up clinics. Furthermore, the benefits of getting the best designed brace in the shortest time may improve the overall effectiveness of the brace treatment because the patient will be using the brace most effectively sooner, during the most beneficial period of rapid adolescent growth. More clinical data are required to truly answer the total benefits of using ultrasound to assist brace casting. The limitation of this method is an experienced ultrasound technician is required during the brace casting to acquire and analyze the data. To overcome this, an automatic ultrasound machine which can scan the back automatically is being considered for future improvements. Also, the custom software developed for the ultrasound imaging measurement needs to be enhanced so that 3D information and automatic measurements can be obtained without requiring significant operator experience.

## Conclusions

The use of the ultrasound system provided a radiation-free method to determine the optimum pressure level and location to obtain the best stimulated in-brace correction during brace casting. Although the long-term results have not yet known the immediate benefits of reduced cost, radiation exposure, and patient impact have merit. The number of radiograph taken per subject was reduced, and the acceptable in-brace correction was attained sooner in the intervention group with less burden on the families and patients.

## Abbreviations

3D: Three dimensional
AIS: Adolescent idiopathic scoliosis
F: Female
M: Male
T: Thoracic
TLSO: Thoraco-lumbo-sacral orthosis
US: Ultrasound
